# The use of small-molecule structures to complement protein–ligand crystal structures in drug discovery

**DOI:** 10.1107/S2059798317000675

**Published:** 2017-02-22

**Authors:** Colin R. Groom, Jason C. Cole

**Affiliations:** a Cambridge Crystallographic Data Centre, 12 Union Road, Cambridge CB2 1EZ, England

**Keywords:** small molecules, conformation, interactions, solubility, Cambridge Structural Database

## Abstract

Many ligand-discovery stories tell of the use of structures of protein–ligand complexes, but the contribution of structural chemistry is such a core part of finding and improving ligands that it is often overlooked. More than 800 000 crystal structures are available to the community through the Cambridge Structural Database (CSD). Individually, these structures can be of tremendous value and the collection of crystal structures is even more helpful. This article provides examples of how small-molecule crystal structures have been used to complement those of protein–ligand complexes to address challenges ranging from affinity, selectivity and bioavailability though to solubility.

## Abstract 2

Small-molecule crystal structures are of tremendous value in understanding protein–ligand complexes, both individually and as a collection.

## 1. Introduction   

Combining macromolecular crystallography with small-molecule crystallography yields valuable complementary information on any ligands that are present, information which can help to address many of the challenges in generating small molecules for use in a biological context. At its simplest, the availability of a small-molecule crystal structure, from either powder diffraction or single-crystal diffraction studies, gives insight into a potential conformation of a molecule. Beyond this, it reveals the interactions that a molecule makes, both with itself and neighbouring molecules. Contrast and comparison of these conformations and interactions with those made with a protein are often revealing.

The value of small-molecule structures extends far beyond the use of *individual* structures. Even when a structure of the same molecule both bound to a protein and in a small-molecule lattice is not available, information from large numbers of related structures is of tremendous value. It may seem somewhat curious to the reader that an article describing how crystal structures of small molecules can complement those of macromolecules is at all necessary. Indeed, in 1971 practitioners of macromolecular and small-molecule crystallography were informed that the Cambridge Crystallographic Data Centre and Brookhaven National Laboratory were to jointly launch a ‘repository system for protein crystallo­graphic data’ to complement the existing system for small-molecule crystal structures (Protein Data Bank, 1971 ▸). The total holding was to be distributed as one.

However, the two communities did begin to drift apart. Crystallographers focused on biological macromolecules tend to be aligned with the biological sections of their institutions, with small-molecule crystallographers typically found in chemistry departments. This division is, perhaps, reinforced by the separation of the output of these scientists into the CSD and the PDB and into journals specializing in one or the other area. Crystallographic societies provided an environment whereby practitioners of these subdisciplines would unite; however, the establishment of discipline-specific special interest groups mitigates some of the success of these.

As a result, different community norms have become established, for example regarding the expectations on reviewers and the release of data. Moreover, the synergies gained from an understanding of macromolecular and small-molecule structures are sometimes overlooked. This article uses a number of examples from the literature to illustrate the impact of combining insights from small-molecule and macromolecular studies. However, to begin with, it is probably worth restating a few of the differences between small-molecule crystal structures (molecules of the type that may be protein ligands) and the structures of macromolecules.

The most significant differences relate to the ratio of observed parameters to modelled variables. In small-molecule crystallography, it is not unusual to have an order of magnitude more measured reflections than modelled parameters. Furthermore, the effective resolution to which small-molecule structures are determined (although ‘resolution’ is not often explicitly referred to by the small-molecule community) is significantly higher: almost all structures are, effectively, determined at atomic resolution, typically described by macromolecular crystallographers as 1.2 Å. Indeed, many are determined at what the macromolecular community would describe as ultrahigh resolution (0.8 Å). To avoid termino­logical tangles, some macromolecular structures have been described as being determined with small-molecule accuracy (Deacon *et al.*, 1997 ▸).

The combination of the factors of high resolution and a high ratio of observations to parameters means that geometrical restraints are seldom used in the refinement of small-molecule structures, so the resulting structures contain less bias towards these restraints. In fact, the restraints commonly used for macromolecular refinement (Engh & Huber, 1991 ▸; Moriarty *et al.*, 2016 ▸; Vagin *et al.*, 2004 ▸) all have their origins in small-molecule structures.

Small-molecule structures are much less dependent on the skilled interpretation of electron density required by those working with macromolecular systems; in most circumstances there is simply one best, correct, model. Solvent molecules are usually well positioned, so that solvent networks can be well understood (Infantes *et al.*, 2003 ▸; Infantes & Motherwell, 2002 ▸). These networks are also observed in high-resolution macromolecular structures and can provide an excellent guide to the placement of solvent molecules when the electron density calculated for a protein–ligand complex is difficult to model. Contrary to common understanding, small-molecule crystals are often not in an anhydrous environment; the environment surrounding a small molecule and the interactions made are reasonably representative of a protein binding site (Boer *et al.*, 2001 ▸; Nissink & Taylor, 2004 ▸; Verdonk *et al.*, 1999 ▸). Disorder is usually resolved into individual positions, particularly where this is over just two sites. Most recent structures include the placement of H atoms. This means that tautomeric states are typically well understood (Cruz-Cabeza & Groom, 2011 ▸) and that the *angular* preferences of hydrogen bonds can be understood (Wood *et al.*, 2009 ▸), something that is seldom commented on in protein–ligand complexes, where undue regard is often given to precise hydrogen *distances*. So many structures are available that the interaction preferences of particular functional groups can be understood by statistical approaches (Taylor, 2014 ▸), allowing the determination of which contacts between atoms do represent genuinely favourable interactions.

### 1.1. The availability of small-molecule crystal structures   

The complete record of every published small-molecule crystal structure determination is available in the Cambridge Structural Database (CSD; Groom *et al.*, 2016 ▸). This contains over 800 000 entries, including many structures published directly through the CSD as a *CSD Communication*. So vast is the number of crystal structures that there are often examples closely related to molecules of interest. Correspondences between CSD entries and ligands bound to macromolecules in structures archived in the Protein Data Bank (PDB; Berman *et al.*, 2000 ▸) are enabled by a CCDC web service that identifies the best representative CSD entry for a given molecule and provides access to its coordinates. Such structures are available for about 1500 PDB ligands in a Chemical Component Model file provided by the PDB (wwPDB, 2015 ▸).

So the world’s collection of existing small-molecule crystal structures is readily available, but what about determining a specific small-molecule crystal structure to complement a protein–ligand complex? Many macromolecular crystallo­graphy groups also have access to small-molecule equipment; indeed, the equipment required and the necessary expertise often already reside in the same institution. It is perhaps down to an underappreciation of their value that small-molecule structures are not determined as a matter of course to accompany a protein–ligand complex. This may be compounded by misunderstandings about the difficulties in obtaining them, particularly relating to compound quantity. However, a straw poll of staff at the Cambridge Crystallo­graphic Data Centre suggested that the minimum amount of material of molecular weight 500, a simple organic compound, needed to stand a reasonable prospect of obtaining a structure in a standard crystallography laboratory, from either a single-crystal or a PXRD experiment, is about 10 mg, an amount that is often within reach, albeit more than the 1–2 mg that might be sufficient to obtain a crystal of a protein–ligand complex.

## 2. Conformations and state   

**Figure 1:**
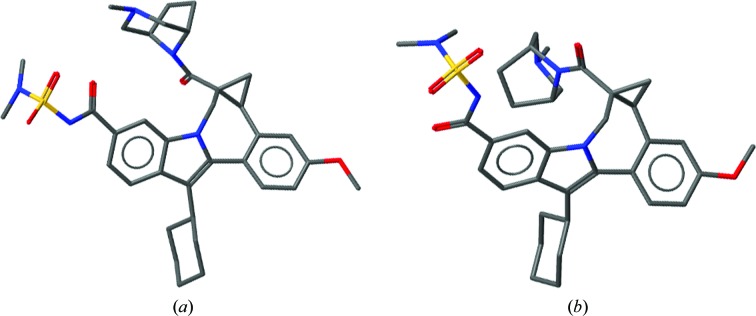
Similarity of single-crystal and protein-bound structures of allosteric HCV NS5B inhibitors. (*a*) Small-molecule structure with CSD Refcode MIYWIC. (*b*) Protein-bound ligand in PDB entry 4nld.

The conformation of a molecule ‘in the solid form’ (*i.e.* a small-molecule single crystal or powder) will be one of a repertoire of conformations that the molecule can adopt. This conformation will be a compromise between the energetic minima of the conformation and the interactions that the molecule can make. Also influencing the conformation may be considerations regarding the generation of symmetry, to allow a repeating lattice and the kinetic accessibility of a particular crystalline form. However, the compromise between conformation and interactions to give either a small-molecule lattice or a protein–ligand complex are fundamentally the same. What are sometimes referred to as the effects of mysterious ‘crystal packing forces’ are in fact both rare and explicable (Cruz-Cabeza *et al.*, 2012 ▸). The beautiful balance seen between adjusting conformation to optimize interactions is common in small-molecule and protein systems. It is usually more appropriate to think of a molecule being gently pulled from its exact energetically minimal conformation to create the best fit to a protein binding site or the most optimal lattice than to think of a molecule being pushed into an unfavourable conformation owing to unfavourable interactions. The maxim that ‘proteins don’t strain ligands, protein crystallographers do’ is worth keeping in mind (Liebeschuetz *et al.*, 2012 ▸; Rupp *et al.*, 2016 ▸). Should the conformation a molecule in a small-molecule lattice be similar to that when bound to a protein, this can give vital information to the structural biologist. An illustrative example is that of HCV NS5B inhibitors developed by Bristol-Myers Squibb (Gentles *et al.*, 2014 ▸). Key to the activity of these molecules are the dihedral angles within the cyclopropylindolobenzazepine rings, specifically the angle between the fused methoxy-substituted phenyl moiety and the indole ring (Fig. 1 ▸). Comparison of the small-molecule structure (CSD Refcode MIYWIC) and protein–ligand complex (PDB entry 4nld) shows this similarity.

**Figure 2:**
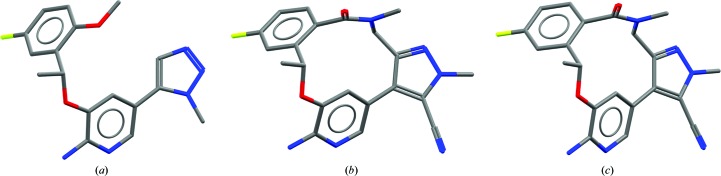
Comparison of single-crystal and protein-bound structures of ALK inhibitors. (*a*) Protein-bound acyclic ligand in PDB entry 4cnh. (*b*) Small-molecule cyclic compound structure with CSD Refcode ZOJLIV. (*c*) Protein-bound cyclic ligand in PDB entry 4cli.

Further examples of similarity in conformation are evident in the development of molecules based on the anticancer agent crizotinib (Johnson *et al.*, 2014 ▸). In the pursuit of compounds with higher affinity towards a clinical mutation of the target kinase, a ligand-cyclization strategy was attempted. Not only did the cyclized compounds produced show almost identical conformations in the small-molecule and protein-bound structures, they superimpose almost perfectly on their acyclic predecessors (Fig. 2 ▸), suggesting that these cyclized compounds need not compromise their geometry in order to form a crystal or to bind to their protein target.

**Figure 3:**
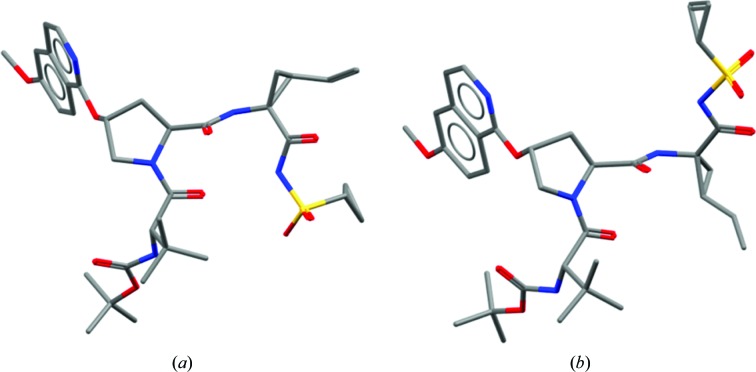
Comparison of single-crystal and protein-bound structures of HCV NS3 inhibitors. (*a*) Small-molecule structure with CSD Refcode MIYWUO. (*b*) Protein-bound ligand in PDB entry 4nwk.

The realisation that there are conformations available to molecules which differ from those observed when bound to a protein can be of enormous impact. For example, initial analysis of the structures of HCV NS3 inhibitors developed by Bristol-Myers Squibb (Scola, Sun *et al.*, 2014 ▸; Scola, Wang *et al.*, 2014 ▸) might suggest properties inconsistent with those most commonly seen in orally bioavailable drugs (Lipinski *et al.*, 1997 ▸). Indeed, reference solely to the protein-bound conformations of these molecules, as shown in Fig. 3 ▸, might suggest they have a rather large polar surface area, with many hydrogen-bonding groups. However, whilst this may be required to bind the target protein (the molecule is, after all, mimicking a peptide), the small-molecule crystal structures show that the molecules have other conformations in their repertoire. These folded conformations demonstrate that the molecule is able to mask some of this polar character and it may be the ability to adopt such a conformation that gives these molecules surprisingly good properties.

## 3. Solubility   

Thus far, we have focused on the influence of conformation on the affinity and properties of molecules. Although reasonably strong binding to a target receptor is indeed important, many other properties influence the use of a compound as a protein binder. Foremost among these is solubility. In a drug-discovery context, solubility influences absorption, bioavailability and target exposure (Di *et al.*, 2009 ▸; Williams *et al.*, 2013 ▸). Poorly soluble compounds may also make it difficult to reconcile the observed *in vitro*, cellular and *in vivo* activities. For the macromolecular crystallographer, the difficulties are no less severe. It can prove difficult to generate protein–ligand complexes, either by co-crystallization or through soaking experiments, for poorly soluble compounds. Even if a compound is more soluble in organic conditions, or with solvents such as DMSO, these can prevent crystallization. It is, therefore, important for the macromolecular crystallographer to understand the drivers behind solubility and, more importantly, to understand how the skills of a structural scientist can be brought to bear on this problem.

Fundamentally, the solubility of a small molecule is determined by its solvation energy and, if crystalline, its lattice energy (Jain & Yalkowsky, 2001 ▸; Wassvik *et al.*, 2008 ▸). Typically, sublimation enthalpies are not available for compounds; therefore, the melting point is often used as a surrogate. It is in the manipulation of this in which structural scientists can play a unique role.

### 3.1. Hydrogen bonding and solubility   

**Figure 4:**
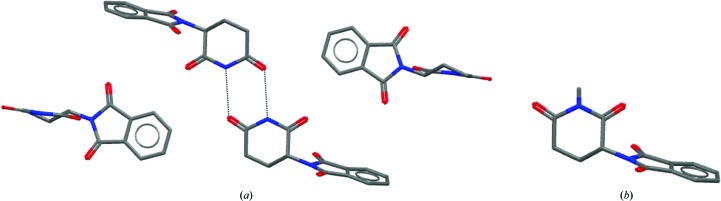
Dimerization in crystals of thalidomide. (*a*) Small-molecule structure with CSD Refcode THALID10. (*b*) *N*-Methyl thalidomide.

One of the best-known examples of how crystal packing influences melting point is the notorious compound thalidomide (Goosen *et al.*, 2002 ▸). As Fig. 4 ▸ shows, thalidomide is able to form hydrogen-bonded dimers between its dioxopiperidine rings, resulting in a crystal with a high melting point of 275°C and a solubility of only 52 µg ml^−1^. *N*-Methyl thalidomide is unable to dimerize in such a manner, resulting in crystals with a lower melting point of 159°C and a corresponding increased solubility of 276 µg ml^−1^.

### 3.2. Stacking and solubility   

**Figure 5:**
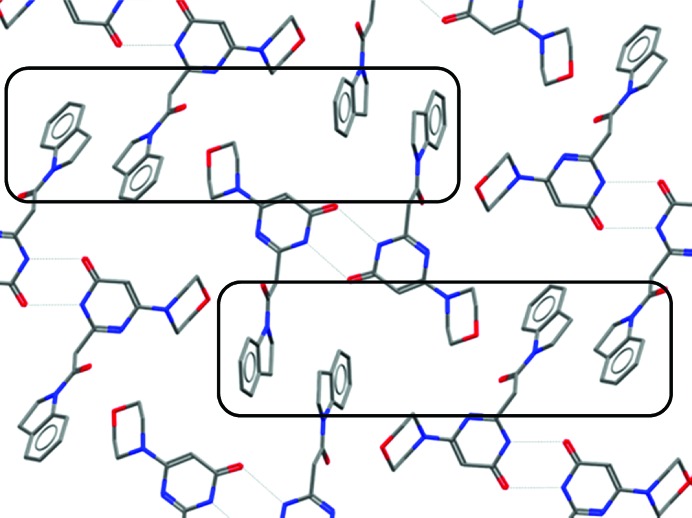
Lattice showing indoline–indoline–morpholine stacking (CSD Refcode TIZLAR).

Stacking interactions between rings in crystal lattices are commonly observed, as exemplified by the indoline-containing structures of the phosphoinositide 3-kinase (PI3K) inhibitors developed by Sanofi (Certal *et al.*, 2014 ▸) and shown in Fig. 5 ▸. There are several examples of compounds containing indoline groups in the CSD. Of these, around a quarter (entries such as ACOBIF, GIMTOM, IQIQII, DCYUD, VIQCEE and QIJDAO) show a self-stacking of their indoline groups. This interaction occurs across a symmetry element, thus providing the two things needed by a molecule for crystallization: self-complementarity and symmetry.

**Figure 6:**
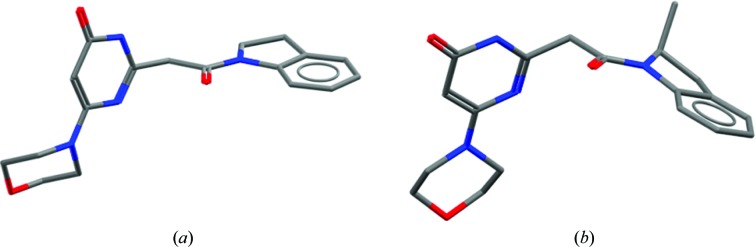
The ffect of methylation of PI3K inhibitors. (*a*) Small-molecule structure with CSD Refcode TIZLAR. (*b*) Protein-bound ligand in PDB entry 4bfr.

Synthesis of substituted indolines, such as those shown in Fig. 6 ▸, prevents such stacking interactions. This change has only a modest effect on the affinity of this compound, but increases the solubility at pH 7.4 to 928 µ*M* from the 12 µ*M* seen in the des-methyl compound, presumably because it is unable to form an equivalent low-energy lattice.

### 3.3. Symmetrical self-recognition and solubility   

**Figure 7:**
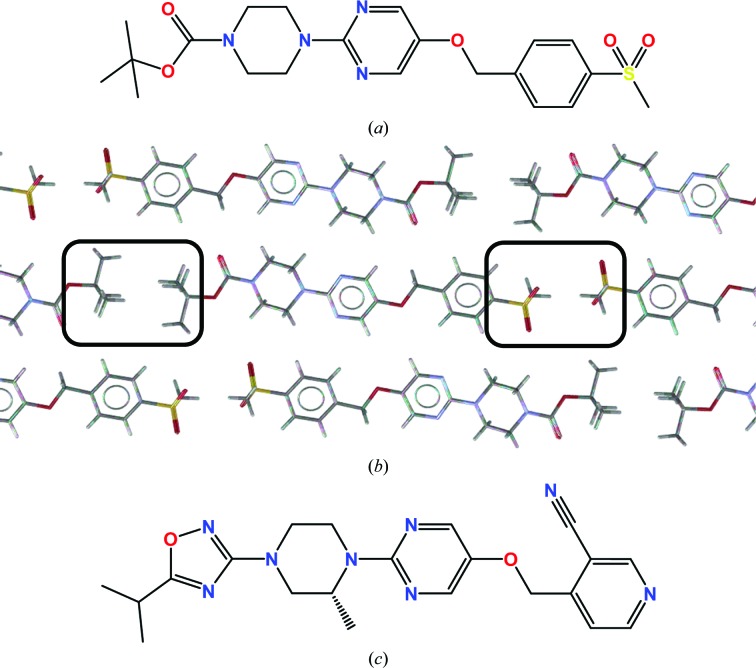
Systematic changes of a GPR119 agonist leading to increased solubility owing to crystal engineering. (*a*) Solubility 0.03 µ*M*. (*b*) Self–self association of terminal groups of molecule *A* in the structural lattice of CSD Refcode SEMPAD. (*c*) Solubility 6 µ*M*.

In the generation of GPR119 agonists, scientists at AstraZeneca made probably the most elegant use of small-molecule crystal structures to improve the solubility of their compounds yet published (Scott *et al.*, 2012 ▸, 2014 ▸). Noting that both ‘ends’ of their molecules self-associated in a symmetry-generating manner, they replaced these groups with moieties which were bio­isosteric but less self-complementary. Furthermore, they methylated a relatively planar central ring structure to reduce the stacking of this system. Combining these changes increased the solubility of their molecules from 0.03 to 6 µ*M* (Fig. 7 ▸).

## 4. Conclusion   

It is somewhat surprising that, given the commitment in obtaining a crystal structure of a protein–ligand complex, more resources are not invested in generating small-molecule crystal structures of these ligands. At its very simplest, this is an effective way of confirming the precise chemical structure of the material thought to be under study. Whether commercially sourced or not, the question ‘is this what it says on the bottle?’ should always be considered (Halford, 2012 ▸).

A small-molecule structure will give precise target values and suggest ranges within which the geometry of a ligand may be restrained during refinement. A small-molecule structure will reveal whether the conformation sampled is the same as in the protein–ligand complex, giving information about the likely entropic components of binding and geometrical compromises made by the ligand, which can be complemented by further computational study. Finally, the interactions made by a small molecule when packing, including with solvent, can be compared with those made in a protein–ligand complex, suggesting where ligand modification may be considered.

In the absence of a small-molecule crystal structure of the precise compound under study, or even a closely related compound, analysis of the vast *collection* of all crystal structures can still be of tremendous value. Knowledge bases such as *Mogul* (Bruno *et al.*, 2004 ▸) allow one to assess whether the proposed geometry of a bound ligand agrees with expectations of molecular geometry from hundreds of thousands of structures. This might give insight into the catalytic mechanism of an enzyme, or might simply suggest that an alternative interpretation of the electron density might be more plausible.

The molecules in the CSD make many millions of unique interactions to form crystalline lattices. Interactions between a protein and ligand simply represent a subset of these, so tools such as *IsoStar* (Bruno *et al.*, 1997 ▸), a knowledge base of such interactions, can be used to verify that a proposed fit of a ligand to electron density results in plausible interactions. Tools such as this can also be used to suggest modifications to increase the affinity of ligand.

Whilst protein–ligand complexes are of undoubtable help in suggesting structural modifications to a ligand that might improve binding, small-molecule structures are of much more value in understanding how one might improve the solubility of a molecule. The insights that these give into the degree of self-recognition of a small molecule are invaluable.

To conclude, the ideal crystallographic data package to understand the properties of a ligand consists of a protein–ligand complex, an apo structure of the protein target and a small-molecule structure of the ligand. Too frequently, research is based solely on the former, not only leading to errors but also reducing the effectiveness of a researcher. Those tasked with reviewing articles describing protein–ligand structures should expect to see convincing reasons why scientists choose to work with only a subset of data. On a more positive note, the skills of the protein crystallographer are entirely suited to understand and exploit what small-molecule crystal structures tell them; it is just a matter of listening.
